# Supplementary material for: The Shigella flexneri effector IpaH1.4 facilitates RNF213 degradation and protects cytosolic bacteria against interferon-induced ubiquitylation
Source: bioRxiv. 2025 Jul 30:2024.09.05.611450. Originally published 2024 Sep 5. Preprint. [Version 2] doi: 10.1101/2024.09.05.611450 (PMC11398459; doi:10.1101/2024.09.05.611450)
Supplement: Supplement 1 [file NIHPP2024.09.05.611450v2-supplement-1.pdf]

bioRxiv preprint doi: https://doi.org/10.1101/2024.09.05.611450; this version posted July 30, 2025. The copyright holder for this preprint (which was not certified by peer review) is the author/funder, who has granted bioRxiv a license to display the preprint in perpetuity. It is made available under aCC-BY 4.0 International license.

SUPPLEMENTARY FIGURE LEGENDS

Fig. S1 Addition of an N-terminal HA-tag significantly reduces targeting of 7KR ubiquitin to *S. flexneri* Δ*mxiE*. N-terminally untagged and N-terminally HA-tagged versions of the internally Strep-tagged Ubiquitin (INT-Ub) 7KR mutant variant were expressed in A549 cells. Cells were primed with 100 U/ml IFNγ overnight and co-localization of INT-Ub with cytosolic *S. flexneri* (Sf) Δ*mxiE* was assessed at 4 hpi. An unpaired t-test was conducted. **p<0.01

Fig. S2 Ubiquitin-Activated Interaction Trap (UBAIT) identifies mouse Rnf213 as likely interaction partner of IpaH2.5. (A) Diagram showing the experimental procedure for UBAIT detection of IpaH2.5 and IpaH3 (control) substrates from the colon and caecum of mice infected with *Salmonella enterica* Typhimurium (S. Tm). **(B)** Mass spectrometry of hits identified in the Strep-IpaH2.5 UBAIT (left) as compared to Strep-IpaH3 UBAIT (right). The top 12 proteins identified in the Strep-IpaH2.5 UBAIT samples as determined by peptide abundance are shown. The corresponding peptide abundances from Strep-IpaH3 UBAIT are shown. RNF213 and the previously identified IpaH2.5 substrate RNF31 (HOIP) are highlighted. PSM = peptide spectrum match. ND = Not Detected. NR = Not Reported.

Fig. S3 Deletion of *ipaH1.4* but not *ipaH2.5* results in RNF213 recruitment to cytosolic *S. flexneri*. (A) A549 cells were primed with IFNγ (100 U/ml) and infected with the indicated strains expressing GFP for 4 hours at an MOI of 100. RNF213 recruitment to cytosolic bacteria was quantified. **(B)** IFNγ-primed A549 RNF213^{KO} cells were infected with the indicated PilT^{+} *S. flexneri* strains for 3 hours at an MOI of 25 and HOIP protein levels were assessed by Western blotting. **(C)** Representative microscopy images showing anti-M1-linked ubiquitin staining of Δ*ipaH1.4 S. flexneri* bacteria in IFNγ-primed WT A549 cells. Quantified data are shown in Fig. 5E. Percentages of WT and Δ*ipaH1.4 S. flexneri* bacteria stained with anti-K27-linked ubiquitin **(D)** or anti-K63-linked ubiquitin **(E)** in IFNγ-primed WT A549 cells. **(F)** Percentages of anti-M1-linked ubiquitin positive Δ*ipaH1.4 S. flexneri* bacteria in IFNγ-primed WT, RNF213^{KO}, HOIL-1^{KO}

bioRxiv preprint doi: https://doi.org/10.1101/2024.09.05.611450; this version posted July 30, 2025. The copyright holder for this preprint (which was not certified by peer review) is the author/funder, who has granted bioRxiv a license to display the preprint in perpetuity. It is made available under aCC-BY 4.0 International license.

and HOIP^{KO} A549 cells. Data are represented by the mean ± SEM from three independent experiments. An unpaired t-test was conducted for **(D, E)**. Data are represented by the mean ± SEM from three independent experiments. Two-way ANOVA with Tukey's multiple comparison tests were performed for **(A,F)**; all statistically significant comparisons are shown. **** $p<0.0001$; ***$p<0.001$; **$p<0.01$; ns, not significant

Fig. S4 Mouse Rnf213 binds to Δ*ipaH1.4 S. flexneri* and facilitates linear ubiquitylation of cytosolic bacteria

(A) Immunoblotting for mouse Rnf213 protein expression in untreated and IFNγ-primed WT and IFNγ-primed Rnf213^{KO} MEF cells. Each lane represents MEFs from separate mouse embryos that were used for infections. **(B and C)** MEF cells were primed with mouse IFNγ (100 U/ml) and infected with the indicated strains expressing GFP and PilT for 2 hours at an MOI of 100. Rnf213-*S. flexneri* colocalization percentages were quantified and are shown in **(B)**. **(C)** Percentage of M1-linked ubiquitin positive *S. flexneri* in IFNγ-primed WT and Rnf213^{KO} MEF cells. All data are represented by the mean ± SEM from at least three independent experiments. An unpaired t-test was performed in **(B)**. Two-way ANOVA with Tukey's multiple comparison tests were performed in **(C)**. **$p<0.01$; **** $p<0.0001$

bioRxiv preprint doi: https://doi.org/10.1101/2024.09.05.611450; this version posted July 30, 2025. The copyright holder for this preprint (which was not certified by peer review) is the author/funder, who has granted bioRxiv a license to display the preprint in perpetuity. It is made available under aCC-BY 4.0 International license.

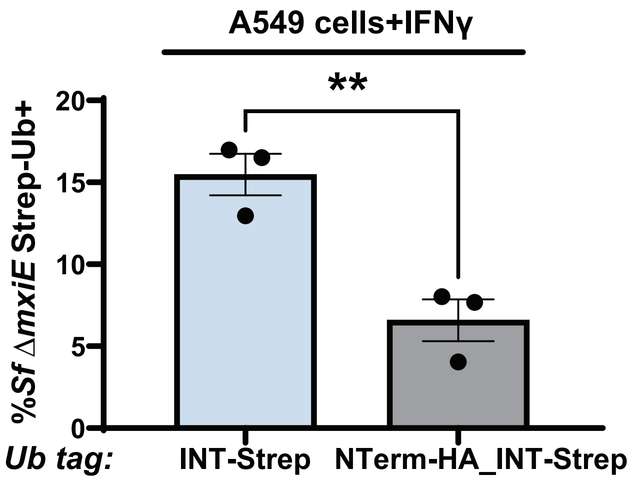

bioRxiv preprint doi: https://doi.org/10.1101/2024.09.05.611450; this version posted July 30, 2025. The copyright holder for this preprint (which was not certified by peer review) is the author/funder, who has granted bioRxiv a license to display the preprint in perpetuity. It is made available under aCC-BY 4.0 International license.

A

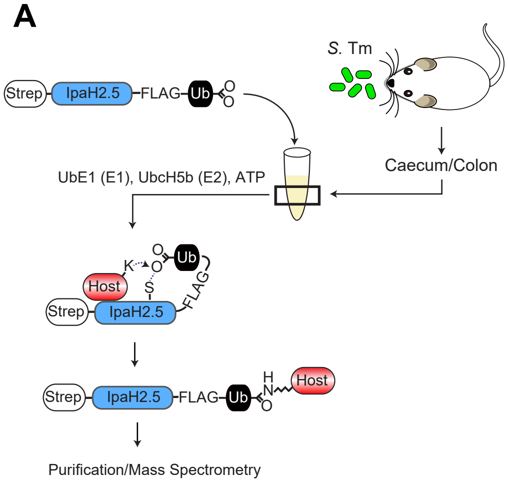

B

Gene	Accession	Molecular Weight	Strep-IpaH2.5 UBAIT				Strep-IpaH3 UBAIT			
			Abundance	Number of Peptides	Number of PSMs	Percent Coverage	Abundance	Number of Peptides	Number of PSMs	Percent Coverage
**Rnf213**	**E9Q555**	**584.3**	**3.51E+07**	**157**	**174**	**37**	**8.33E+04**	**4**	**4**	**1**
Ubr4	A2AN08	571.9	2.14E+06	29	29	9	4.56E+04	4	4	1
Rnf31	Q924T7	119.2	4.33E+06	28	30	36	ND	ND	ND	ND
Trim25	Q61510	71.7	8.36E+06	21	24	39	1.96E+04	1	1	3
Dtx3l	Q3UIR3	83	4.62E+06	18	23	36	6.62E+04	2	2	3
Scyl2	Q8CFE4	103.3	2.37E+06	16	19	26	6.67E+05	10	10	15
Tuba1c	P68373	49.9	2.45E+06	16	36	53	8.93E+05	15	31	44
Rbck1	Q9WUB0	57.5	1.04E+06	14	14	36	ND	ND	ND	ND
Ddx17	Q501J6	72.4	1.47E+05	8	13	15	NR	7	10	12
Ubqln1	Q8R317	62	1.15E+06	8	9	18	2.29E+04	4	4	10
Mvp	Q9EQK5	95.9	2.88E+05	8	8	11	1.41E+05	4	4	6
Birc2	Q62210	69.6	4.02E+05	8	8	13	ND	ND	ND	ND

bioRxiv preprint doi: https://doi.org/10.1101/2024.09.05.611450; this version posted July 30, 2025. The copyright holder for this preprint (which was not certified by peer review) is the author/funder, who has granted bioRxiv a license to display the preprint in perpetuity. It is made available under aCC-BY 4.0 International license.

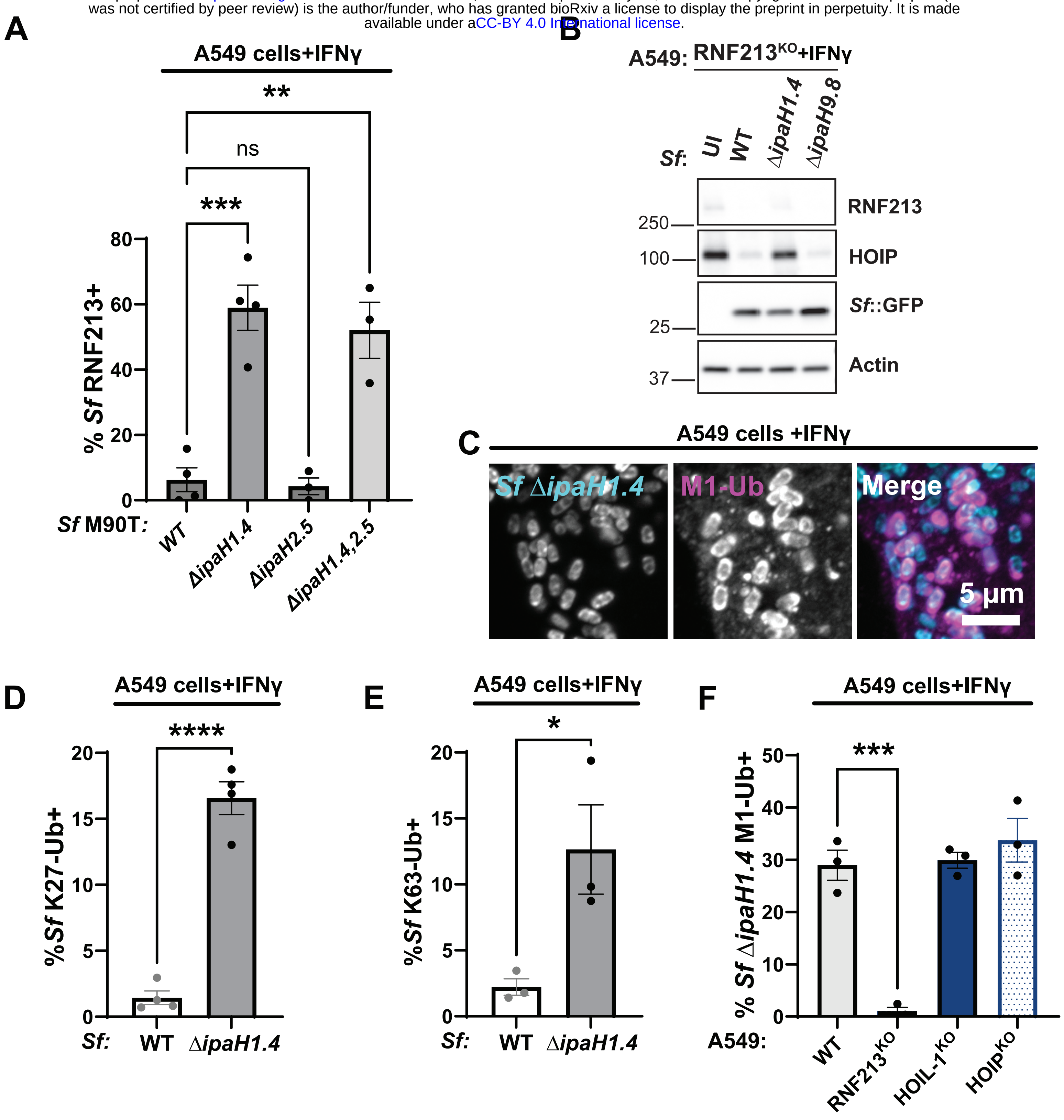

bioRxiv preprint doi: https://doi.org/10.1101/2024.09.05.611450; this version posted July 30, 2025. The copyright holder for this preprint (which was not certified by peer review) is the author/funder, who has granted bioRxiv a license to display the preprint in perpetuity. It is made available under aCC-BY 4.0 International license.

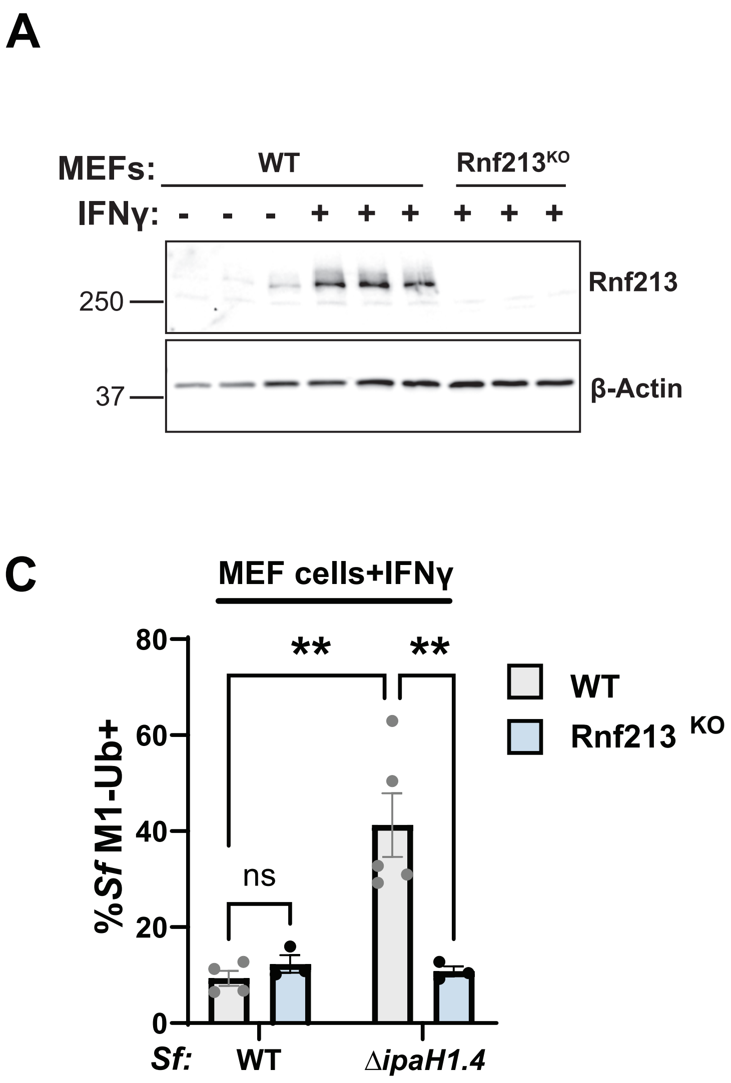

bioRxiv preprint doi: https://doi.org/10.1101/2024.09.05.611450; this version posted July 30, 2025. The copyright holder for this preprint (which was not certified by peer review) is the author/funder, who has granted bioRxiv a license to display the preprint in perpetuity. It is made available under aCC-BY 4.0 International license.

INT-Ub Construct	Amino acid sequence (ORF)
WT	MQIFVKTLTGKTITLEVEPSDTIENVKAKIQDKEGIPPDQQRLIFAGKQLEDGS AWSHPQFEKSATLSDYNIQKESTLHLVLRLRGG
7KR	MQIFVRTLTGRTITLEVEPSDTIENVRARIQDREGIPPDQQRLIFAGRQLEDGS AWSHPQFEKSATLSDYNIQRESTLHLVLRLRGG
6KR+K6	MQIFVKTLTGRTITLEVEPSDTIENVRARIQDREGIPPDQQRLIFAGRQLEDGS AWSHPQFEKSATLSDYNIQRESTLHLVLRLRGG*
6KR+K11	MQIFVRTLTGKTITLEVEPSDTIENVRARIQDREGIPPDQQRLIFAGRQLEDGS AWSHPQFEKSATLSDYNIQRESTLHLVLRLRGG*
6KR+K27	MQIFVRTLTGRTITLEVEPSDTIENVKARIQDREGIPPDQQRLIFAGRQLEDGS AWSHPQFEKSATLSDYNIQRESTLHLVLRLRGG*
6KR+K29	MQIFVRTLTGRTITLEVEPSDTIENVRAKIQDREGIPPDQQRLIFAGRQLEDGS AWSHPQFEKSATLSDYNIQRESTLHLVLRLRGG*
6KR+K33	MQIFVRTLTGRTITLEVEPSDTIENVRARIQDKEGIPPDQQRLIFAGRQLEDGS AWSHPQFEKSATLSDYNIQRESTLHLVLRLRGG*
6KR+K48	MQIFVRTLTGRTITLEVEPSDTIENVRARIQDREGIPPDQQRLIFAGKQLEDGS AWSHPQFEKSATLSDYNIQRESTLHLVLRLRGG*
6KR+K63	MQIFVRTLTGRTITLEVEPSDTIENVRARIQDREGIPPDQQRLIFAGRQLEDGS AWSHPQFEKSATLSDYNIQKESTLHLVLRLRGG*
5KR+K27-63	MQIFVRTLTGRTITLEVEPSDTIENVKARIQDREGIPPDQQRLIFAGRQLEDGS AWSHPQFEKSATLSDYNIQKESTLHLVLRLRGG*

Table 1: Internally STREP-tagged Ubiquitin (INT-Ub) constructs. Ubiquitin lysines are shown in red. Strep tag sequence in highlighted in green
